# Hypercalcemia after transplant nephrectomy in a hemodialysis patient: a case report

**DOI:** 10.1186/1752-1947-1-164

**Published:** 2007-12-03

**Authors:** Ivo Quack, Magdalena Woznowski, Gisela Schieren, Stefan M Weiner, Guido Winnekendonk, Faruk Tokmak, Lars C Rump, Dirk Rattensperger

**Affiliations:** 1 Department of Nephrology, Marienhospital Herne, Ruhr-University Bochum, Hoelkeskampring 40, 44625 Herne, Germany; 2 Department of Radiology, Marienhospital Herne, Ruhr-University Bochum, Hoelkeskampring 40, 44625 Herne, Germany; 3 Kuratorium für Heimdialyse und Nierentransplantation, Cruismannstrasse 37, 44807 Bochum, Germany; 4 current address : Renal department, Heinrich-Heine University, Duesseldorf, Germany; 5 current address : Internal Medicine 2, Nephrology, Krankenhaus Bamherzige Brueder, Trier, Germany

## Abstract

**Introduction:**

Hypercalcemia is a complication often seen in chronic hemodialysis patients. A rare cause of this condition is sarcoidosis. Its highly variable clinical presentation is challenging. Especially in patients suffering chronic kidney graft failure the nonspecific constitutional symptoms of sarcoidosis like fever, weight loss, arthralgia and fatigue may be easily misleading.

**Case presentation:**

A 51 year old male developed hypercalcemia, arthralgia and B-symptoms after explantation of his kidney graft because of suspected acute rejection. The removed kidney showed vasculopathy and tubulointerstitial nephritis, which had not been overt in the biopsy taken half a year earlier. Despite explantation and withdrawal of the immunosuppression the patient's general condition deteriorated progressively. A rapid rise in serum calcium finally provoked us to check for sarcoidosis. CT scans of the lungs, broncho-alveolar-lavage and further lab tests confirmed the diagnosis.

**Conclusion:**

This case demonstrates that withdrawal of immunosuppressive drugs sometimes unmasks sarcoidosis. It should be considered as differential diagnosis even in hemodialysis patients, in whom other reasons for hypercalcemia are much more common.

## Introduction

Hypercalcemia is a complication often seen in chronic hemodialysis patients. In these patients various circumstances can cause a high serum calcium level. Most of them can be detected easily like a tertiary hyperparathyreoidism, the use of 1α-hydroxylated vitamin D metabolites, the inappropriate medication with calcium phosphate binders or the use of a high dialysate calcium [1].

The challenge for the physician is to recognize the rare causes of hypercalcemia in hemodialysis patients such as non-hodgkin lymphoma, multiple myeloma or sarcoidosis as an example of granulomatous diseases.

Knowing that symptomatic sarcoidosis is usually treated with immunosuppressive drugs [2], it is easy to understand that the withdrawal of immunosuppression in transplanted patients no longer prevents the "de novo" development or the recurrence of sarcoidosis. Up to date literature counts only very few cases of sarcoidosis appearing shortly after withdrawal of immunosuppressive regimen, all of them recurrent forms of sarcoidosis [3-8]. In contrast to these reports, our case describes most likely a "de novo" sarcoidosis already appearing after reduction of the immunosuppressive regimen.

## Case presentation

**Figure 1:**
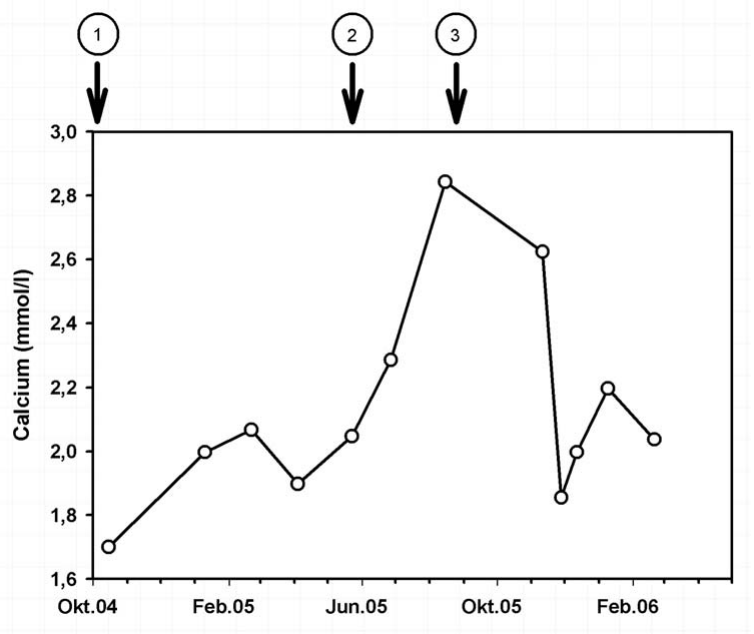
Course of serum calcium (mmol/l). Arrow 1: reduction of immunosuppression to 5 mg prednisolone per day. Arrow 2: complete stop of immunosuppression. Arrow 3: restart of prednisolon 50 mg per day.

**Figure 2:**
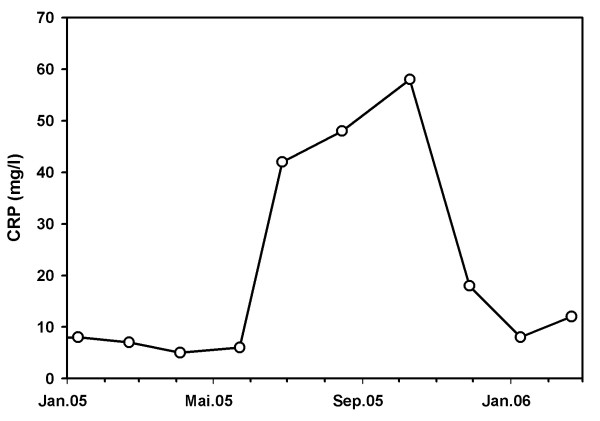
Course of c-reactive protein (mg/l)

A 51-year-old man developed end-stage renal failure due to biopsy proven IgA nephritis. He started hemodialysis in April 1977. 1980, 1985 and 1997 he received cadaveric kidney transplantation. In 1998 a parathyreoidectomy was performed because of a severe tertiary hyperparathyreoidism. From 1997 to November 2003 the patient did not need dialysis. In December 2003 hemodialysis was started again due to declining kidney function. Transplant biopsy rendered chronic transplant vasculopathy. The graft remained in situ to preserve residual diuresis. The immunosuppressive regimen was reduced to 5 mg prednisone and 50 mg azathioprine per day until October 2004. From this point onwards only 5 mg prednisone per day was given. In June 2005 the patient presented with an inflammatory syndrome with slightly elevated body temperature (up to 37.5°Celsius) and a general weakness of the body. To rule out acute rejection the patient underwent another transplant biopsy which besides the known transplant vasculopathy revealed tubulointerstitial nephritis as a new finding. This was interpreted as acute rejection, the graft was removed (end of June 2005) and immunosuppression was completely withdrawn. A few days after a moderate fever reappeared. The patient complained about testicular pain. Ultrasound showed a picture resembling epididymitis. An empiric treatment with ciprofloxacin did neither alleviate the complaints nor the ultrasound aspects of the enlarged epididymis. In the following weeks the patient's general condition deteriorated progressively. He complained about insomnia and strong pruritus. This was accompanied by unwanted loss of 4 kg body weight within 12 weeks. Alternating arthralgia was the most bothering symptom. X-rays and ultrasound examinations of the affected joints ruled out specific affections or diseases of the bones. Laboratory tests showed a rapidly increasing serum calcium: 2.84 mmol/l (normal value: 2.2–2.65 mmol/l), (fig. 1) despite normal or only moderately elevated serum levels of parathyroid hormone (PTH) (PTH intact: 1.2–4.5 pmol/l). Serum alkaline phosphatase was elevated with a level of 173 U/l (normal value: 35–104 U/l). C-reactive protein was only moderately elevated (fig. 2). We changed the patient to a low calcium dialysate concentration and to the phosphate binder sevelamer. Vitamin D substitution had been stopped more than three months before. Despite all these measures the high serum calcium level and the clinical symptoms remained unchanged.

**Figure 3:**
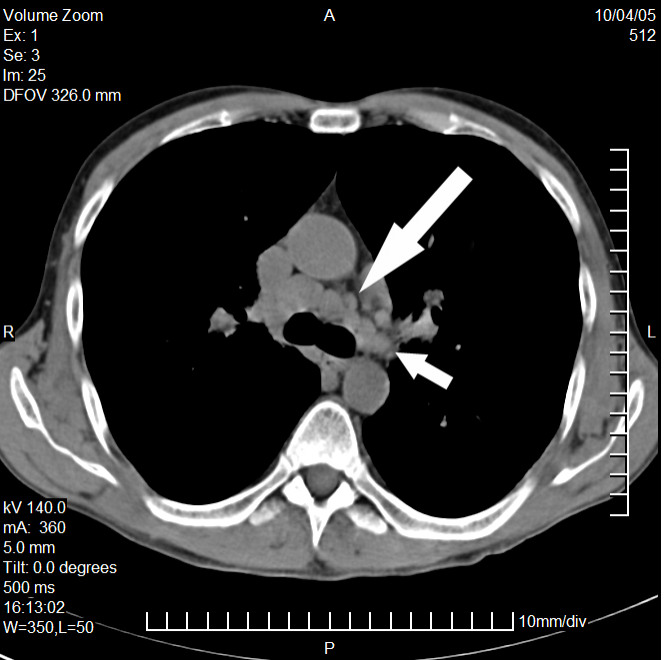
Computed tomography of the chest (I). CT of the chest: Distinct mediastinal (large arrow) and perihilar lymphadenopathy (small arrow)

**Figure 4:**
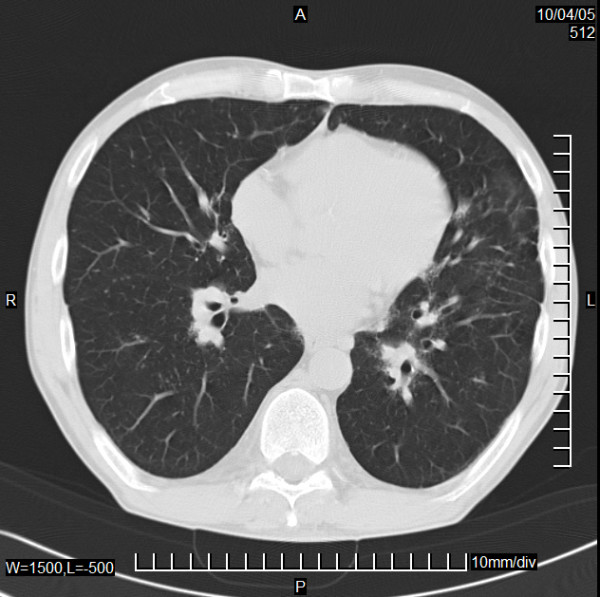
Computed tomography of the chest (II). CT of the chest: Nodular and reticular interstitial lung involvement.

Finally the persisting hypercalcemia provoked us to check for sarcoidosis. Indeed X-rays of the thorax revealed a pathological hilus configuration. A thoracic CT scan showed mediastinal and hilar lymphadenopathia of 2–3 cm in diameter and a disseminated, micronodulary shaped densities of both lungs (Fig. 3 and 4).

A transbronchial biopsy rendered only chronic-atrophic bronchitis. But broncho-alveolar lavage (BAL) revealed a CD4/CD8 ratio above the normal value. The diagnosis sarcoidosis was strengthened by further lab tests: Angiotensin-converting enzyme level: 158 U/l (normal value: 20–60 U/l). Soluble Interleukin2 receptor: 4133 U/ml (normal value: 220–710 U/ml) were elevated. We also found high levels for lysozyme, but no sign of a serum hypergammaglobulinaemia.

With the suspected diagnosis of sarcoidosis we decided to administer prednisone at a dose of 50 mg/day for 3 weeks. In the following time the dose was tapered 2.5 mg every week up to a permanent dose of 5 mg/day. In a short time the patient's general condition improved tremendously. Within a few days the body temperature returned to normal values and pain, anorexia and pruritus disappeared. Serum calcium values returned to normal levels (Fig. 1).

The suppressed PTH intact level found back to normal values within 8 weeks. Serum alkaline phosphatase and angiotensin converting enzyme returned to normal under steroid therapy: AP: 79 U/l, normal: 35–104 U/l, ACE: 54 U/L, normal: 20–60 U/l. In a CT scan in April 2006 the micronodulary alterations of both lungs were no longer seen. Number and size of the affected mediastinal lymphnodes were markedly reduced.

Bodyplethysmography still showed a restrictive alteration. We administered an inhalative salmeterol-fluticason combination. In April 2006 the pulmonary function was completely intact. No signs of restriction or obstruction were found in the tests.

For more than one year the patient is now without clinical and biological symptoms taking 5 mg alternating 2.5 mg prednisone per day.

## Conclusion

Sarcoidosis is a multisystem disorder of unknown etiology characterized by the accumulation of lymphocytes, mononuclear phagocytes and non-caseating granuloma in involved tissues [9]. Due to the frequency of pulmonary involvement bronchoscopy is an important diagnostic tool. Bronchoscopy enables the investigator to identify endobronchial lesions, which can be found in almost half of the cases [10]. In our patient tissue biopsy did not show signs of sarcoidosis, but broncho-alveolar lavage rendered a lymphocytic alveolitis with a CD4/CD8-ratio (>3.5) which is highly consistent with sarcoidosis. The sensitivity of CD4/CD8-ratio ranges from 42 to 59% and specificity from 76–96% [10]. Furthermore, it can be speculated if the tubulointerstitial nephritis was a sign of renal involvement and therefore responsible for graft failure. The true incidence of sarcoidosis kidney disease is hard to define. Notably granulomatous interstitial nephritis accounts only for 10–15% of renal involvement. Interstitial nephritis without granuloma has been described but seems much less common [11]. In our case histology showed significant interstitial infiltration, moderate tubulitis and intimal arteriitis. Glomeruli had a normal morphology and no granulomata were found. Altogether, sarcoidosis cannot be ruled out, but sarcoid interstitial nephritis without granulomata is reported to be very rare, so that rejection seems to be more likely in our patient. However, the assumed epididymitis was probably a sarcoid lesion. In an autopsy series, 5% of cases had genital involvement (most frequently of the epididymis), with bilateral disease present in approximately one-third of them. It is often asymptomatic, but patients may also present with a scrotal mass or pain. Testicular granulomata are much less common [12]. Finally it was only the synopsis of the clinical manifestations, the pathologic lab tests (hypercalcemia, ACE, IL-2-Receptor, lysozyme) and finally the response to treatment with steroids which allowed to confirm the diagnosis.

Our patient received an immunosuppressive treatment for a long time and retrospectively it is difficult to say whether sarcoidosis came up "de novo" after withdrawal of azathioprin and prednisone or if it was reactivated. IgA nephropathy was reason for end stage renal disease in our patient thirty years ago. The association of sarcoidosis and IgA glomerulonephritis is uncommon but has been reported [13]. Unfortunately we had no possibility to re-examine the patient's initial renal biopsy to look for typical signs of sarcoidosis. Neither the patient's history offers any signs for sarcoidosis before, nor did a pulmonary CT scan three years earlier. Thus we assume that sarcoidosis developed "de novo" after reduction of the immunosuppressive regimen and was finally aggravated when the medication was stopped completely. There are only a few reports describing sarcoidosis in kidney graft recipients after withdrawal of immunosuppression (see Table 1). The spectrum of clinical presentation is highly various and reached from high fevers, neurologic symptoms, graft failure and lung involvement including pleural effusion [3-7]. Distinct hypercalcemia was only reported in two patients. That should remind us, that many cases do not present with massive hypercalcemia. A calcium level in the upper normal range or slightly above that should also catch our attention. In patients with end stage renal disease tertiary hyperparathyreoidism, the use of vitamin D metabolites, calcium phosphate binders or high dialysate calcium are frequent causes of hypercalcemia and should be considered first. Further workup should follow a standardized regimen, like the one proposed by Carroll and colleagues [14]. Noteworthy, all patients developed most likely a reactivation of sarcoidosis. In every case sarcoidosis occurred either after steroids have been tapered or in a patient receiving a steroid-free immunosuppressive regimen from beginning. This underlines the important role of steroids in the treatment of sarcoidosis. Hypercalcemia and the severity of clinical symptoms provoked us to initiate systemic treatment using prednisone immediately. Various theories of the pathogenesis (infection, hypersensitivity and autoimmunity) exist. Sarcoidosis may result from an exposure to an antigen in a genetically predisposed, susceptible host [2,11]. When therapy is required, corticosteroids are considered standard [15]. Studies demonstrating their ability to modify the long-term outcome in this disease are still lacking. Often, the adverse side effects of corticosteroids necessitate the addition of other immunomodulating substances [15]. Our patient remained on low-dose steroids without any clinical symptoms more that one year after diagnosis. This case demonstrates that withdrawal of immunosuppressive drugs sometimes unmasks sarcoidosis. It stresses the diagnostic difficulties of sarcoidosis due to its highly variable clinical presentation. Moreover, it reminds every nephrologist to consider sarcoidosis as a differential diagnosis even in hemodialysis patients, in whom other reasons for hypercalcemia are much more common.

**Table 1:** Cases reporting sarcoidosis after reduction of immunosuppression

Shen et al. Am J Med. 1986 Apr;80(4):699–702.	Reactivation of sarcoidosis in kidney graft	[7]
Brown et al. Nephrol Dial Transplant. 1992;7(2):173.	Reactivation after transplantation (steroid-free immunosuppressive regimen)	[3]
Herrero et al. Nephrol Dial Transplant. 1998 Dec;13(12):3280–1.	Reactivation in kidney graft after steroid withdrawal, Hypercalcemia	[4]
Schmidt et al. Transplantation. 1999 Nov 15;68(9):1420–3	Reactivation after steroid withdrawal (lung, pleura), Hypercalcemia	[6]
Kukura et al. Nephrol Dial Transplant (2004) 19: 1640–1642	Reactivation in graft after steroid withdrawal (during pregnancy)	[5]

## Competing interests

The author(s) declare that they have no competing interests.

## Authors' contributions

IQ was involved in patient's care, revising the manuscript and creating the figures, MW was involved in patient's care, GS and SMW were responsible for patient's management, GW was responsible for diagnostics and provided CT scans; LCR was involved in drafting and critically revising the manuscript, DR was responsible for collecting data, drafting and revising the manuscript. All authors have read and approved the final manuscript.

## Consent

Written informed consent has been obtained for publication.
